# DNA-Launched Alphavirus Replicons Encoding a Fusion of Mycobacterial Antigens Acr and Ag85B Are Immunogenic and Protective in a Murine Model of TB Infection

**DOI:** 10.1371/journal.pone.0136635

**Published:** 2015-08-28

**Authors:** Neha Dalmia, William B. Klimstra, Carol Mason, Alistair J. Ramsay

**Affiliations:** 1 Department of Microbiology, Immunology and Parasitology, LSU Health Sciences Center, New Orleans, Louisiana, United States of America; 2 Department of Microbiology and Molecular Genetics, University of Pittsburgh, Pittsburgh, Pennsylvania, United States of America; 3 Department of Microbiology, Immunology and Parasitology, LSU Health Sciences Center, New Orleans, Louisiana, United States of America; University of Delhi, INDIA

## Abstract

There is an urgent need for effective prophylactic measures against *Mycobacterium tuberculosis* (*Mtb*) infection, particularly given the highly variable efficacy of Bacille Calmette-Guerin (BCG), the only licensed vaccine against tuberculosis (TB). Most studies indicate that cell-mediated immune responses involving both CD4+ and CD8+ T cells are necessary for effective immunity against *Mtb*. Genetic vaccination induces humoral and cellular immune responses, including CD4+ and CD8+ T-cell responses, against a variety of bacterial, viral, parasitic and tumor antigens, and this strategy may therefore hold promise for the development of more effective TB vaccines. Novel formulations and delivery strategies to improve the immunogenicity of DNA-based vaccines have recently been evaluated, and have shown varying degrees of success. In the present study, we evaluated DNA-launched Venezuelan equine encephalitis replicons (Vrep) encoding a novel fusion of the mycobacterial antigens α-crystallin (Acr) and antigen 85B (Ag85B), termed Vrep-Acr/Ag85B, for their immunogenicity and protective efficacy in a murine model of pulmonary TB. Vrep-Acr/Ag85B generated antigen-specific CD4+ and CD8+ T cell responses that persisted for at least 10 wk post-immunization. Interestingly, parenterally administered Vrep-Acr/Ag85B also induced T cell responses in the lung tissues, the primary site of infection, and inhibited bacterial growth in both the lungs and spleens following aerosol challenge with *Mtb*. DNA-launched Vrep may, therefore, represent an effective approach to the development of gene-based vaccines against TB, particularly as components of heterologous prime-boost strategies or as BCG boosters.

## Introduction

A third of the human population is latently infected with *Mycobacterium tuberculosis* (*Mtb*), and at risk for disease reactivation [1]. The World Health Organization global TB report for 2014 estimated 9 million new TB cases and 1.5 million TB-related deaths in 2013 [2]. Increases in the incidence of TB are fueled by infections caused by multi-drug resistant and extensively-drug resistant *Mtb* strains, while, co-infection with HIV-1 is the most common cause of immune suppression in latently infected individuals, raising the risk of TB disease from a 10% lifetime chance to 10% annually [3]. Although *Mycobacterium bovis* BCG, the only licensed TB vaccine, is widely used for routine childhood vaccinations, its variable efficacy in adults and concerns with its use in HIV-infected persons warrants the development of novel and more effective prophylactic vaccines for controlling the TB epidemic [1,4–6]. Immune correlates of vaccine-mediated protection against *M*. *tuberculosis* infection are not yet well defined, however most studies indicate that T helper type 1 (Th1) cell-mediated immune responses mediated by CD4+ and CD8+ T cells are important components of an effective immune response to TB infection [7–10].

DNA vaccination has been used to induce humoral and cellular immunity, including CD4+ and CD8+ T-cell responses against a variety of bacterial, viral, and parasitic antigens, and affords some protective immunity against these pathogens [11,12]. However, conventional DNA vaccines are poorly immunogenic in larger mammals, including primates and humans [13–16]. DNA vaccines have been clinically tested in vaccine trials against HIV and malaria, but to date, they have not entered clinical trials as candidate TB vaccines [17–19]. New formulation and delivery strategies to improve their immunogenicity have recently been evaluated, including the use of “DNA-launched” replicons derived from alphaviruses [20–24]. Alphaviruses are arthropod-borne members of the family *Togaviridae* with a positive-sense single-stranded RNA genome. Replicons based on different alphaviruses, including Sindbis virus (SINV), Semliki Forest virus (SFV) and Venezuelan equine encephalitis virus (VEEV), have recently been developed as a vaccine vector platform [24–31]. Replacement of structural genes downstream of the internal 26S promoter with a transgene of interest gave rise to virus-replicon particles (VRP) expressing high levels of the gene product, while the particles themselves appear to target dendritic cells [32,33], activate a variety of innate inflammatory factors [34,35] and induce apoptosis [36,37], all attractive features for vaccination. However, potential safety issues and problems with large-scale manufacture of alphavirus VRP have hampered their further development. The recent development of self-amplifying replicons “launched” from DNA plasmids addresses these issues, while providing DNA-based vaccines of greatly enhanced immunogenicity [24–29]. These constructs are essentially DNA vaccines where a cDNA copy of the viral replicase and the vaccine antigen are placed on a plasmid backbone under the control of a cytomegalovirus promoter [28]. They retain the immune-stimulatory qualities of VRP and have proven to be immunogenic in several murine models of infection [24–29].

In the present study, we have engineered DNA-launched VEE replicons (Vrep) encoding a fusion of two mycobacterial antigens, Ag85B and α-crystallin (Acr). Ag85B is an immunogenic mycolyl transferase involved in the coupling of mycolic acids with arabinogalactan and is necessary for cell wall formation [38]. Acr is a 16kDa homolog of α-crystallin family of heat shock proteins and is a part of the 48-gene DosR (regulator of dormancy) regulon under the control of the transcription factor DosR [39,40]. Acr is widely regarded as a “latency associated antigen”, but appears to be immunogenic soon after infection in the mouse model (Mehta and Ramsay, unpublished data). We have evaluated the immunogenicity and protective efficacy of these constructs in a mouse model of acute pulmonary TB infection. Our studies indicate that Vrep induce long-lasting antigen-specific T cell responses in both the spleen and in pulmonary tissues, and reduce bacterial loads in these tissues following aerosol challenge with virulent *Mtb*.

## Material and Methods

### Vaccine vectors

A gene fusion encoding Acr (GenBank Acc No. M76712.1) upstream of Ag85B (GenBank Acc. No. X62398) was codon-optimized using the Java Codon Optimization Tool (http://www.jcat.de) and was manufactured by GenScript (Piscataway, New Jersey).

The methodology for construction of pHis DNA vaccines has been described elsewhere [41]. For construction of pHis-Acr/Ag85B (DNA-Acr/Ag85B), the Acr/Ag85B gene fusion was PCR-amplified from pUC57-Acr/Ag85B and subcloned into the DNA vaccine vector-pHis that contains CpG motifs in the vector backbone (Coley Pharmaceutical Group, Wellesley, Massachusetts) as a BamHI/EcoRI fragment using T4 DNA Ligase (Invitrogen, Carlsbad, California). The ligation product was transformed into competent TOP10 E. coli cells (Invitrogen) and positive clones were confirmed by PCR using vector-specific primers and DNA sequencing. pHis vectors without Acr/Ag85B gene inserts were used as control DNA vaccines. DNA vaccines were prepared in bulk for *in vivo* experiments using the Endo-free plasmid Mega kit (Qiagen, Gaithersburg, Maryland) according to the manufacturer’s instructions, and were resuspended in 0.85% sterile saline.

The Venezuelan equine encephalitis virus (VEEV) DNA plasmid replicon (pVrep) was constructed using overlap PCR to fuse the 5’ end of the Trinidad Donkey strain replicon vector, pVR21 [42,43] to the estimated RNA transcriptional start sequence of the cytomegalovirus Immediate Early gene promoter in the pCMV-Tag cloning vector (Agilent Technologies). This generated an amplicon of 1.37 kb containing the entire 700bp CMV promoter and 670 bp of the 5’ end of the replicon which was subsequently cloned into the pCR-Blunt vector using the ZeroBlunt cloning Kit (Agilent Technologies). Using HpaI and NotI, the 3’ end of the pVR21 replicon was then cloned into the pCR-blunt vector, creating the Vrep replicon launched by the CMV promoter.

The firefly luciferase (Luc), green fluorescent protein (GFP) or Acr/Ag85B genes were cloned into the pCR-Blunt vector as AscI/PacI fragments using the ZeroBlunt cloning kit and then transferred into pVrep via Asc I and Pac I sites as previously described [44], giving rise to Vrep-Luc, Vrep-GFP, and Vrep-Acr/Ag85B respectively. To confirm protein expression, BHK cells were electroporated with 20 μg of GFP or Acr/Ag85B pVrep using a BioRad electorporator (200 mV, 1000μF resistance). At 18–24 hrs post electroporation, cells were lysed using whole cell lysis buffer and 20–30μg separated by SDS-PAGE followed by western blot for FLAG Tag as previously described [44,45].

### Animals

Specific-pathogen-free mice were purchased from Charles River (Raleigh, North Carolina) and housed in the Louisiana State University Health Sciences Center (LSUHSC) animal care facility. 6–8 wk old female Balb/c mice were used in all experiments. BCG vaccinated and/or *M*. *tuberculosis*-challenged mice were housed in a Biocontainment Level-3 (BSL-3) Laboratory operated in accordance with the appropriate safety precautions recommended by the Centers for Disease Control and Prevention and monitored by the LSUHSC Institutional Biosafety Committee.

### Ethics Statement

All procedures involving mice were approved by the Louisiana State University Health Sciences Center Institutional Animal Care and Use Committee (IACUC), Approval Number 3010. LSUHSC Animal Care is fully accredited by AAALAC. All invasive procedures were performed under anesthesia with a mixture of KetaThesia (ketamine HCl, 100 mg/mL, Butler Animal Health Supply, Dublin, Ohio) and xylazine (10 mg/mL, Henry Schein, Mandeville, Louisiana) diluted eight-fold in phosphate-buffered saline (PBS; Gibco, Invitrogen, Carlsbad, California). Mice were euthanized by cervical dislocation under anesthesia.

### Immunization

Animals were immunized with Vrep or conventional DNA vaccines at the time points indicated in Table 1, with specific details in figure legends. For immunization, 10ug of Vrep or 30ug of conventional pHis DNA vaccine were given by an intramuscular (IM) injection into each tibialis muscle (20ug Vrep or 60ug total DNA respectively in phosphate-buffered saline (PBS)) followed by immediate electroporation with 5 pulses at 150 V using an ECM 830 Electroporation System and caliper electrode (BTX–Harvard Apparatus, Holliston, Massachusetts). These vaccine doses were previously shown, by IFNγ ELISPOT assay, to be optimal in mice for immune priming of CD4+ T cells for subsequent boosting with recombinant adenovirus vaccines encoding homologous vaccine antigens (**ref 41**, data not shown).

**Table 1:** Immunization Protocols.

Group	Week 0	Week 3 or 6
DNA Vaccination	DNA-control	DNA-Acr /Ag85B
	DNA-Acr/Ag85B	DNA-Acr /Ag85B
Vrep immunization	Vrep-Luc	Vrep-Luc
	Vrep-Luc	Vrep-Acr/Ag85B
	Vrep-Acr/Ag85B	Vrep-Acr/Ag85B

DNA vaccines were administered IM as described in Materials and Methods, such that each animal received a total of 60μg of designated vaccine at week 0 and again at week 3. Vrep vaccines were given IM as described in Materials and Methods, such that each animal received a total of 20μg of designated vaccine at week 0 and again at week 3 or week 6. See figure legends for specific details within each experiment.

### Isolation of mononuclear cells

At 3 or 10 wk post-immunization, animals were euthanized, and spleens were isolated by gross dissection. Tissues were processed into single-cell suspensions in complete medium (CM) consisting of RPMI 1640 supplemented with 20 mM L-glutamine, 10 mM HEPES, 50 ug/mL streptomycin, 50 U/mL penicillin, 50 mM 2-mercaptoethanol (Sigma–Aldrich, St. Louis, Missouri) and 10% FCS (Hyclone, Thermo Fisher Scientific, Waltham, Massachusetts). All reagents were purchased from Gibco unless otherwise specified. Red blood cell lysis was performed using RBC Lysing Buffer (Sigma–Aldrich). Lung tissues were also harvested, minced in sterile 25ml beakers, and incubated in complete digestion medium (CDM) in a 37° C water bath prior to processing into single-cell suspensions as described above. CDM consisted of CM without FCS, and with addition of 1mg/ml collagenase I and 30ug/ml DNase I (Worthington Biochemical Corp, Lakewood, NJ).

### Peptides

Synthetic peptide oligomers representing CD4+ and CD8+ T cell epitopes in Acr and Ag85B were used to stimulate antigen-specific responses in IFN-γ ELISpot and intracellular cytokine staining assays. The peptide sequences were as follows: Acr CD4+ T cell epitope RDGQLTIKAERTEQKDFDGRS [46,47], Ag85B CD4+ T cell epitope HSWEYWGAQLNAMKGDLQ [48], Ag85B CD8+ T cell epitope MPVGGQSSF [49], and Acr CD8+ T cell epitope TYDKGILTV (Mehta and Ramsay, unpublished). All peptides were synthesized by Genscript (Piscataway, NJ).

### Interferon gamma (IFN-γ) EliSPOT Assay

IFN-γ ELISpot was performed using 96-well Multiscreen TM-IP plates (Millipore, Billerica, Massachusetts) and Mabtech reagents for ELISpot assay for mouse interferon-γ (Mariemont, Ohio) according to manufacturer’s protocols and as described elsewhere [41]. Cells were stimulated with CD4+ or CD8+ peptides at a final concentration of 5 ug/mL. Spots were developed using BCIP/NBT substrate (Moss Substrates, Pasadena, Maryland) and counted with an AID-ELISPOT counter (AutoImmun Diagnostika GmbH, Strasburg, Germany). Data are presented as spot forming cells (SFCs) per million cells.

### Intracellular cytokine staining (ICS)

Two million lymphocytes were seeded into 96 well round bottomed plates (Corning Enterprises, Corning, New York) and stimulated with 5 ug/mL peptides at 37° C for 2 hours, then treated with BD GolgiPlug Protein Transport Inhibitor (BD Pharmigen, San Diego, California) at 0.1 uL/well, followed by incubation at 37° C for an additional 4 hours. Control wells without peptide restimulation were established within in each experiment. Cells were then stained with anti-CD3, anti-CD4 and anti-CD8 antibodies, washed, fixed and permeabilized using BD CytoFix/Cytoperm Fixation/Permeabilization Kit (BD Biosciences, San Diego, California), and then stained for intracellular expression of IFN-γ, TNF-α and IL-2. Fluorochrome antibodies used for staining included CD3e-Pacific Blue, CD4-FITC, CD8-PE-Cy5, IFN-γ-APC, IL-2-PE, and TNF-α-PE-Cy7 (BD Pharmigen). 400,000 events were acquired on a BD LSR II flow cytometer and data were analyzed using FlowJo software version 8.8.6 (Tree Star, Ashland, Oregon). For analysis, lymphocyte populations were initially identified by forward-scatter (size) and side-scatter (granularity) profiles. Lymphocytes positive for CD3 were subsequently sorted into CD3+CD4+ and CD3+CD8+ subsets, prior to the measurement of cytokine secretion. Multifunctional T cell subsets were identified using the Boolean gating feature in FlowJo.

### Aerosolized *M*. *tuberculosis* challenge

*Mtb* strain H37Rv (ATCC No. 27294, Rockville, MD) was grown in Middlebrook 7H11 broth at 37°C for 14 days. The culture was concentrated by centrifugation, gently sonicated at 95 W for 10 seconds in a cup-horn sonicator, and stored in aliquots at -80°C after titration. At the time of inoculation, an aliquot was thawed, gently sonicated, and diluted in endotoxin-free PBS at 2 x 10^6^ colony-forming units (CFU)/ml. For the challenge experiment at 10 wk after their second immunization with Vrep-Acr/Ag85B or DNA-Acr/Ag85B, mice were given *Mtb* by the aerosol route in a Glas-Col inhalation exposure system (Glas-Col, LLC, Terre Haute, IN). Exposure times were calibrated to deliver 50–100 CFU of bacteria into the lungs of each infected mouse. Control mice were given 1x10^5^ CFU of BCG, or were mock-immunized with saline, subcutaneously via the footpad at 3 wk or 10 wk prior to *Mtb* challenge. Mice were sacrificed at 6 wk after *Mtb* challenge, and bacterial loads in the lungs and spleens were determined by serial dilution of tissue homogenates on 7H11 agar plates in quadruplicate. The plates were incubated at 37°C for 21 days in sealed plastic bags prior to counting bacterial colonies.

### Statistical analysis

The unpaired two-tailed Student’s t-test was used to determine statistical significance of all data. P-values of 0.05 or less were considered significant.

## Results

### Confirmation of expression of Acr/Ag85B fusion by Vrep

**Fig 1:**
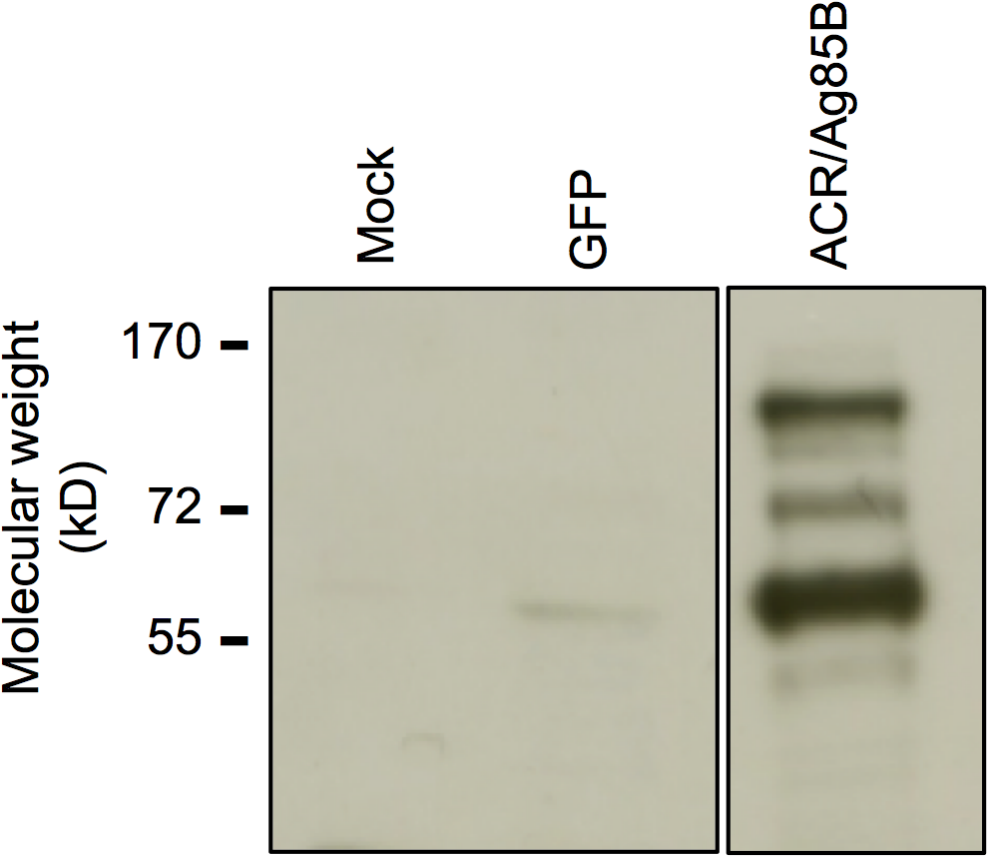
Expression of Acr/Ag85B fusion protein in Vrep-Acr/Ag85B confirmed by Western blot. BHK cells were electroporated with 15μg of plasmid replicons or were not electroporated (mock), lysates were prepared 18 hours later, and a Western blot was performed using anti-FLAG antibody as described in Materials and Methods.

As shown in Fig 1, expression of the Acr/Ag85B fusion by Vrep-Acr/Ag85B was confirmed by Western blot using a FLAG tag. The band for the fusion protein was seen, as expected, at approximately 55kD. The presence of multiple gel bands is sometimes seen with replicon expression, since the replicons themselves can affect macromolecular synthesis within transfected cells. The presence of multiple bands in the ACR/Ag85 lanes could reflect these effects. In addition, due to a high level of expression from the subgenomic promoter of the replicon, certain forms/complexes of the protein may also be expressed that have previously not been detected.

### Vrep encoding *Mtb* fusion antigen generates sustained antigen-specific T cell responses

**Fig 2:**
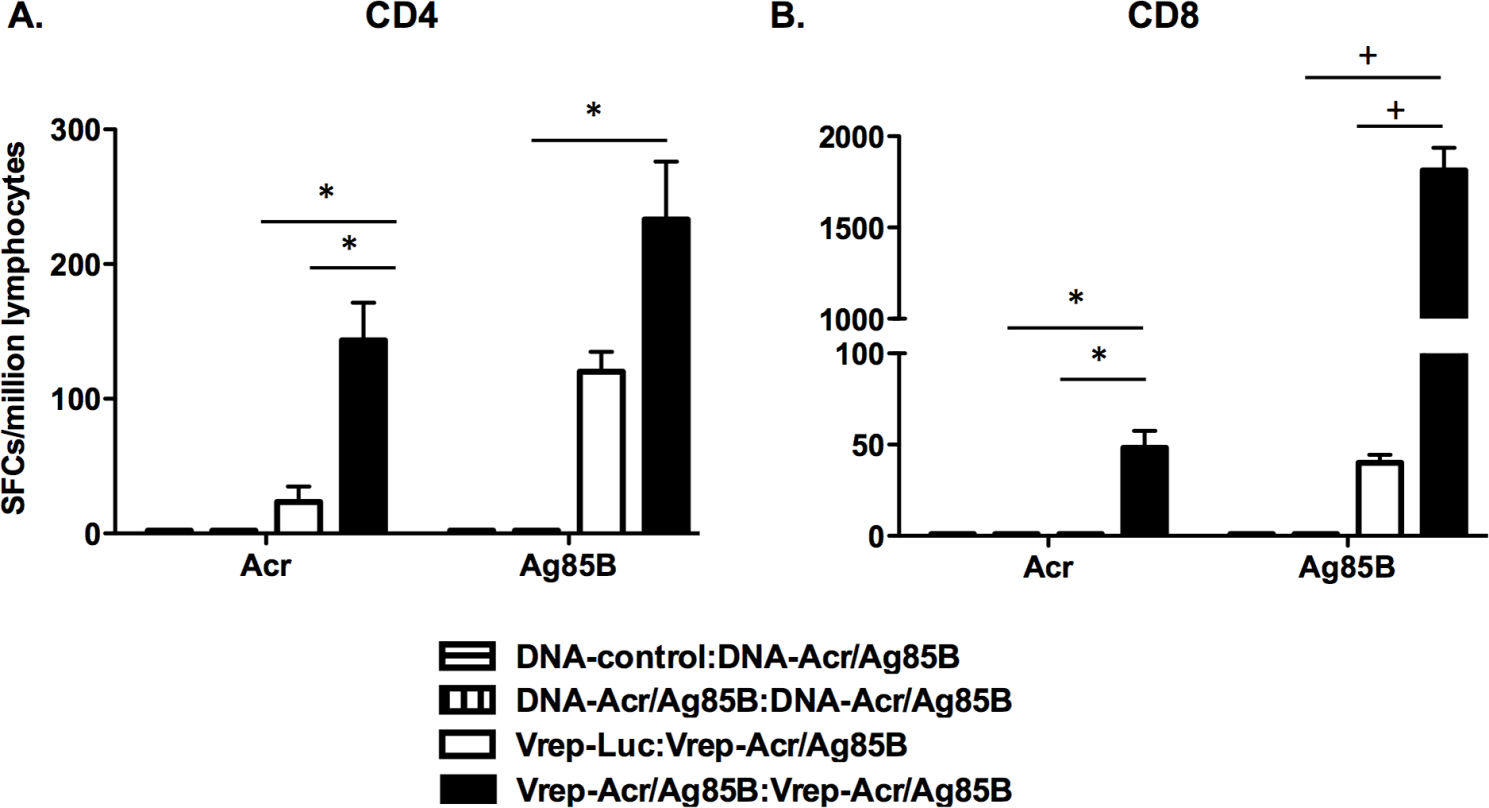
Vrep-Acr/Ag85B is immunogenic for CD4+ and CD8+ T cell responses. Mice were immunized twice at weeks 0 and 3 with Vrep or conventional DNA vaccines as described in Materials and Methods and shown in Table 1. The different immunization groups are denoted by the initial vaccine given at week 0 followed by the second vaccine given at week 3 (eg. DNA-control:DNA-Acr/Ag85B). Splenocytes were harvested at 3 wk post-immunization and both Acr and Ag85B-specific CD4+ (A) and CD8+ (B) T cell responses were assessed by IFN-γ ELISpot assay. Data shown are mean counts of spot forming cells (SFCs) ± SEM from pooled splenocyte samples (n = 5) in a single experiment and are representative of three such experiments, * p < 0.05, + p < 0.01, by Students t-test.

Initial experiments were designed to evaluate the immunogenicity of DNA launched Vrep vaccines encoding the Acr/Ag85B fusion antigen. Mice immunized with either one or two doses of Vrep-Acr/Ag85B generated strong CD4+ T and CD8+ T cell responses in the spleen as measured in IFN-γ ELISPOT assays (Fig 2). Significantly higher numbers of Acr-specific CD4+ and CD8+ T cells producing IFN-γ were observed following a second dose of Vrep-Acr/Ag85B, while approximately 2-fold higher numbers of Ag85B-specific CD4+ T cell responses, and far greater numbers of Ag85B-specific CD8+ T cells were seen after two doses (Fig 2). These data show a clear boosting effect of Vrep. T cell responses to both of the antigens encoded by the vaccine were evident after priming and boosting, demonstrating the immunogenicity of the Acr/Ag85B fusion. Negligible responses were detected following vaccination with conventional DNA vaccine encoding the fusion antigen, despite the capacity of these constructs to prime for antigen-specific CD4+ and CD8+T cell responses upon subsequent boosting with recombinant adenovirus vectors that also encoded Acr/Ag85B (data not shown).

**Fig 3:**
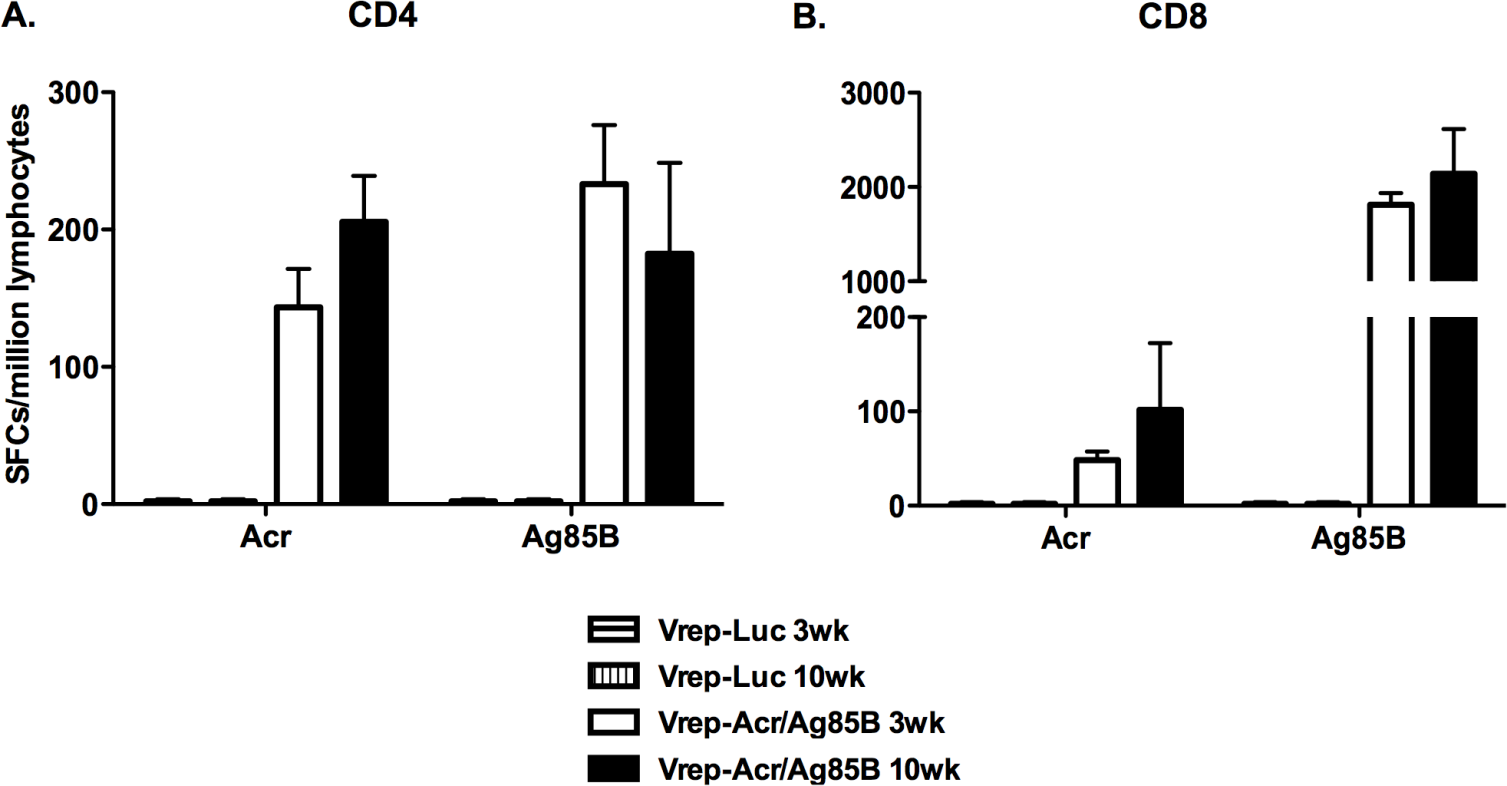
CD4+ and CD8+ T cell IFN-γ responses induced by Vrep vaccination persist at least up to 10 weeks post immunization. Mice were immunized twice at weeks 0 and 3 with Vrep-Luc or Vrep-Acr/Ag85B vaccines as described in Materials and Methods and shown in Table 1. Splenocytes were harvested at 3 or 10 weeks post immunization and Acr and Ag85B-specific CD4+ (A) and CD8+ (B) T cell responses were assessed by IFN-γ ELISpot. Data shown are mean counts of spot forming cells (SFCs) ± SEM from pooled splenocyte samples (n = 5) in a single experiment and are representative of three such experiments. Student’s t-test was used for all group comparisons.

In order to measure the longevity of T cell responses induced by the Vrep fusion, mice were immunized as before, with two doses of vaccine at 3 wk intervals. As shown in Fig 3, in mice given Vrep-Acr/Ag85B, Acr and Ag85B-specific CD4+ and CD8+ T cell IFN-γ responses seen in the spleen at 3 wk post boosting persisted for at least 10 wk, indicative of the generation of sustained T cell responses against both antigens. Negligible responses were seen at 10 wk in mice given DNA-Acr/Ag85B (data not shown), as had been found at the 3 wk time point. The reminder of the study was focused on the immunogenicity and protective efficacy of the Vrep-Acr/Ag85B construct.

**Fig 4:**
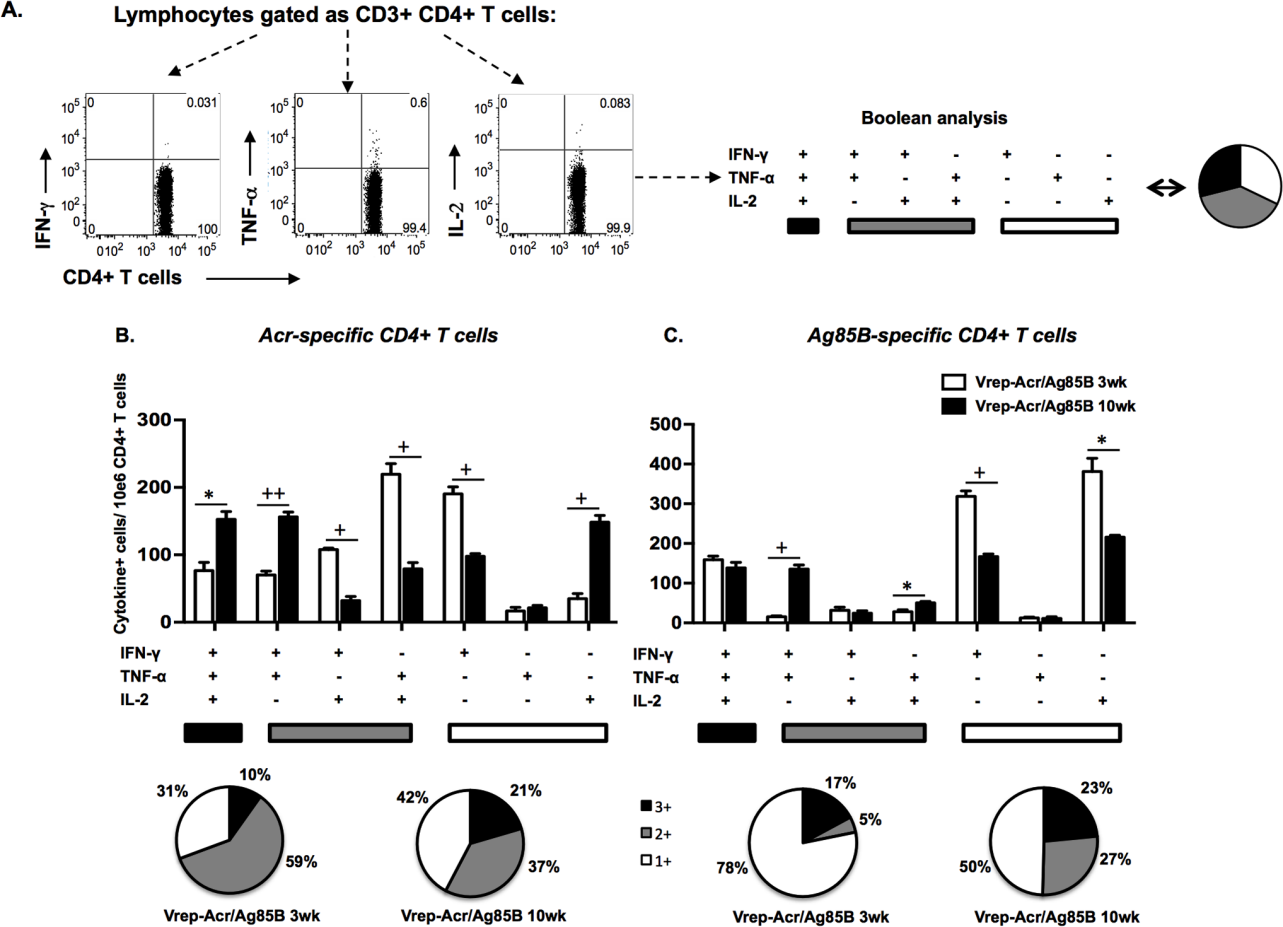
Multifunctional CD4+ T cells persist for at least 10 weeks post immunization with Vreps. Mice were immunized twice at weeks 0 and 3 with Vrep-Luc or Vrep-Acr/Ag85B vaccines as described in Materials and Methods and shown in Table 1. A) Representative dot plots showing lymphocytes gated as CD3+ CD4+ T cells secreting IFN-γ, TNF-α or IL-2 in response to recall antigen. After gating, Boolean analyses allow determination of concurrent expression of these cytokines. Splenocytes were harvested at either 3 or 10 weeks post-immunization and Acr-specific (B) and Ag85B-specific (C) CD4+ T cells producing IFN-γ, TNF-α and/or IL-2 were assessed by ICS after subtraction of values for media controls in each case. Data shown are numbers of cytokine secreting CD4+ T cells per million CD4+ T cells in splenocytes from individual mice (n = 5) as mean ± SEM in a single experiment that is representative of three such experiments. * p < 0.05, + p < 0.01, ++ p < 0.001 by Student’s t-test. Pie charts under the bar graphs depict relative percentages of CD4+ T cells in each vaccine group that produced all three of these cytokines (3+), two of these cytokines (2+) or a single cytokine (1+), after exposure to recall antigen and were created using the SPICEv4.1.5 and PESTLEv1.5.2 software programs (a gift from M. Roederer, Vaccine Research Center, NIAID/National Institutes of Health).

Next, intracellular cytokine staining was performed in order to evaluate numbers of polyfunctional CD4+ and CD8+ T cells that were generated in the spleen following immunization with Vrep-Acr/Ag85B (Fig 4). Consistent with the ELISpot data, Acr-specific (Fig 4B) and Ag85B-specific (Fig 4C) CD4+ T cell responses were found at both 3 wk and 10 wk post-boosting. Acr-specific and Ag85B-specific CD4+ T cell subsets secreting IFN-γ/TNF-α/IL-2 or IFN-γ/TNF-α were particularly evident at the later time point, indicative of a persistent, mature T cell response to Vrep-encoded antigens. Indeed, the relative proportions of responding polyfunctional subsets, and those secreting individual cytokines, by 10 wk after immunization were similar (in the case of Acr) or increased (Ag85B) compared to the profiles seen at 3 wk post-immunization, as shown in the pie-charts under Fig 4B and 4C. Significant numbers of IL-2 secreting CD4+ T cells, either polyfunctional or producing IL-2 alone, were also present at 10 wk after boosting. Antigen-specific CD8+ T cell responses following Vrep immunizations showed a similar profile to their CD4+ T cell counterparts (data not shown). Ag85B or Acr-specific T cell responses were negligible in mice primed with control Vrep-Luc or among cells cultured in the absence of Ag85B or Acr peptides (data not shown).

### Systemic and mucosal immune responses to parenteral Vrep-Acr/Ag85B delivery were not enhanced by extension of the interval between immunizations

**Fig 5:**
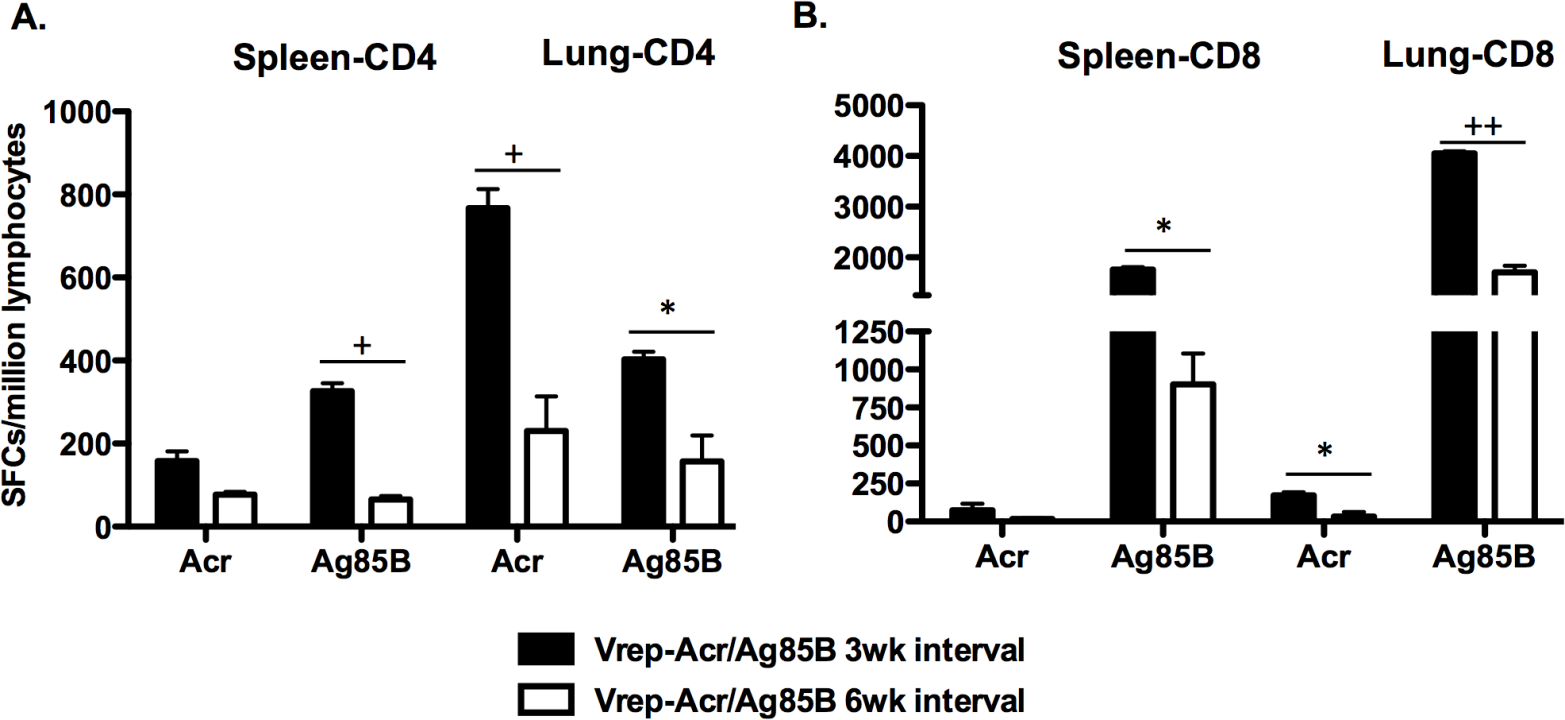
Increasing the time interval between homologous Vrep-DNA immunizations does not increase the magnitude of T cell IFN-γ responses in spleens and lungs. Mice were immunized twice at 3 or 6-week intervals with Vrep vaccines and splenocytes and lung lymphocytes were harvested at 10 weeks after immunization for assay of splenic and pulmonary Acr and Ag85B-specific CD4+ T (A) and CD8+ T (B) cell responses by IFN-γ ELISpot, as described in Materials and Methods and shown in Table 1. Data shown are SFCs/million lymphocytes from pooled splenocytes or lung samples (n = 5) in one experiment that is representative of three such experiments and are shown as mean SFCs ± SEM. * p ≤ 0.05. + p ≤ 0.01. ++ p = 0.001 by Student’s t-test.

In attempts to optimize the outcome of Vrep immunization, we investigated whether increasing the interval between successive doses of Vrep-Acr/Ag85B from 3 wk to 6 wk affected vaccine immunogenicity. Ten wk after the last dose of Vrep in each case, mice were sacrificed to assess antigen-specific CD4+ and CD8+ T cell responses by IFN-γ-ELISpot (Fig 5). The magnitude of Acr-specific CD4+ (Fig 5A) and CD8+ (Fig 5B) T cell responses measured in splenocytes was not significantly affected by increasing the interval to 6 wk, while corresponding Ag85B-specific T cell responses were actually diminished. The opportunity was also taken in this experiment to study vaccine-specific T cell responses in the lungs of mice given Vrep via the i.m. route, the rationale being that local immune responses against *Mtb* are potentially important, given the usual pulmonary route of infection. Interestingly, antigen-specific CD4+ (Fig 5A) and CD8+ (Fig 5B) T cell responses against both Acr and Ag85B components of the vaccine were found in the lungs of systemically immunized mice. Indeed, pulmonary responses were of greater magnitude than corresponding splenic responses in these animals, particularly in the case of CD4+ T cell immunity. It was also clear that increasing the interval between i.m. doses of Vrep from 3 wk to 6 wk diminished vaccine-induced pulmonary CD4+ and CD8+ T cell responses. Further analyses of the numbers of Acr and Ag85B-specific multifunctional CD4+ and CD8+ T cells secreting IFN-γ, TNF-α and IL-2 in lungs and spleens, by intracellular cytokine staining, confirmed these findings (data not shown).

In summary, it was evident that i.m. delivery of Vrep encoding Acr/Ag85B generated strong systemic and pulmonary mucosal CD4+ and CD8+ T cell responses. The development of significant levels of Th1-type T cell immunity in the lung tissues following systemic administration of these constructs is of interest, given the primary route of infection by *Mtb*. It was also clear that increasing the interval between doses of Vrep was of no apparent benefit in terms of the magnitude of resulting systemic or local T cell responses.

### Immunization with Vrep-Acr/Ag85B vaccine protects mice against pulmonary challenge with aerosolized *M*. *tuberculosis*

Given the strong systemic and pulmonary mucosal CD4+ and CD8+ T cell responses generated after i.m. delivery of Vrep, we used a mouse model of pulmonary *Mtb* infection to evaluate the protective efficacy of Vrep-Acr/Ag85B against an aerosol challenge, since Th1-type immune responses are thought to be an important component of protection against *Mtb* infection. Mice were immunized twice with Vrep-Acr/Ag85B at 3 wk intervals and challenged with *M*. *tuberculosis* H37Rv strain 10 wk later. Vehicle (naïve) and BCG-vaccinated mice served as negative and positive control groups respectively. Bacterial loads were enumerated at 6 wk post challenge.

**Fig 6:**
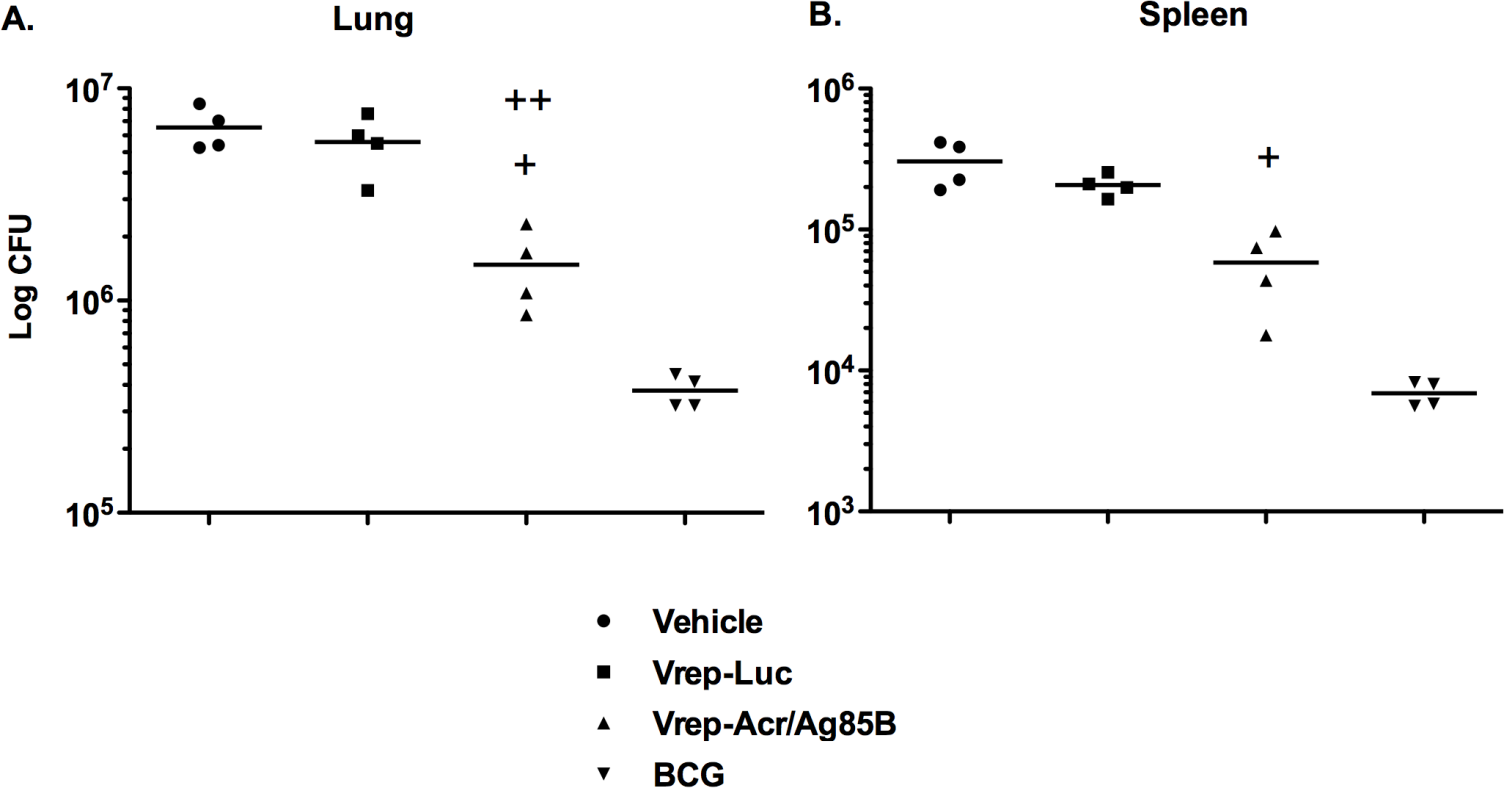
Vrep-Acr/Ag85B vaccine is protective in a murine model of acute *M*. *tuberculosis* infection. Mice were immunized twice with Vrep-Luc or Vrep-Acr/Ag85B at a 3-week interval as described in Materials and Methods and shown in Table 1. BCG and vehicle immunized mice served as positive and negative control groups respectively. Ten weeks after immunization, mice (n = 5) were infected via the pulmonary route with 50–100 CFU of aerosolized *Mtb* and were sacrificed 6 weeks later for assessment of bacterial burden in lungs (A) and spleens (B). Bacterial colonies were counted at 21 days after plating lung or spleen homogenates on 7H11 agar plates. Horizontal lines represent mean CFU for each group. For (A) + p < 0.01 (vs Vrep-Luc), ++ p < 0.001 (vs naïve); for (B) + p < 0.01 (vs Vrep-Luc and vehicle) by Student’s t-test.

As shown in Fig 6, vaccination with Vrep-Acr/Ag85B significantly reduced bacterial loads in the lungs, the primary site of TB infection (Fig 6A), with reductions of the order of 4-fold compared to ‘naïve’ mice given vehicle only or to mice given control Vrep encoding an irrelevant vaccine protein (Vrep-Luc). Since *Mtb* can disseminate to extra-pulmonary tissues following infection, splenic mycobacterial burdens were also quantified to determine the capacity of Vrep immunization to limit systemic growth. Interestingly, Vrep-Acr/Ag85B significantly reduced extra-pulmonary growth of mycobacteria, with burdens in the spleens 5-fold lower than in naïve mice and 4-fold lower than in those given control Vrep. No reductions in bacterial growth were seen in mice immunized with conventional DNA vaccine encoding the Acr/Ag85B fusion antigen (data not shown). Control mice given BCG at either 3 wk or 10 wk (not shown) prior to *Mtb* challenge were protected at similar levels.

In conclusion, our data show that immunization with Vrep constructs encoding Ag85B and Acr via the intramuscular route induced potent antigen-specific CD4+ and CD8+ T cell responses in both lungs and spleens. Pulmonary mucosal T cell responses correlated with a 4-5-fold reduction of *Mtb* growth in immunized mice.

## Discussion

In the present study, we evaluated the immunogenicity and protective efficacy of DNA-launched VEE replicons encoding a novel fusion of the mycobacterial antigens Acr and Ag85B in a murine model of pulmonary TB infection. The Vrep-Acr/Ag85B construct was highly immunogenic, with CD4+ and CD8+ T cell responses against both fusion antigens persisting for at least 10 wk following immunization. Strikingly, when given intramuscularly, Vrep vaccines also generated strong, persistent T cell responses in the pulmonary tissues. This is the first report of which we are aware showing high-level pulmonary CD4+ and CD8+ T cell immunity to encoded vaccine antigens following parenteral administration of alphavirus replicons. Vaccine-induced immunity correlated with a 4 to 5-fold reduction in bacterial loads in pulmonary and extra-pulmonary tissues following aerosol challenge with aerosolized *Mtb*.

Parenteral delivery of immunogens often fails to induce mucosal immune responses [50,51]. The capacity of Vrep to induce vaccine-specific T cell responses, including multifunctional T cell responses, in lung tissues of mice immunized up to 10 wk earlier is a potentially important feature for their further development as a vaccine platform. Multifunctional T cells secreting IFN-γ and IL-2 are thought to correlate with protective efficacy in several disease models, including *Leishmania major* infection in mice [52] and SIV infection in rhesus macaques [53], while their presence in the lungs correlated with protection in a murine model of TB infection [54]. In addition, the absence of polyfunctional CD4+ T cells in the lungs of HIV-infected individuals correlated with a high level of susceptibility to TB infection [55]. While clarification of the protective role of T cell subsets in human TB infection will require further analyses, including longitudinal studies [56,57], the generation of pulmonary immune responses against vaccine-expressed TB antigens by Vrep in the present study is of particular interest, given the primary route of transmission of TB as an aerosol via the lungs, and increasing evidence that local immune defense mechanisms can play an important role in protective immunity against TB [58]. Induction of effective local and circulating immunity against TB would help control bacterial numbers in the lungs, while also preventing or reducing extra-pulmonary growth.

It will be important to clarify mechanisms underlying the induction of pulmonary T cell responses by Vrep in this system. It appears that VRP themselves can act as adjuvants for both systemic and mucosal immune responses when given parenterally [30,31], with up regulation of expression of the mucosal homing receptor α4β7 on antigen-specific T cells in peripheral draining lymph nodes correlating with increased numbers of these cells in the mucosal tissues [31]. Further studies will be required to ascertain a role for α4β7, or other mucosal homing cell surface integrins such as VLA-4, in the mucosal immunogenicity of the DNA-based Vrep vaccine.

Previous studies using DNA-based Sindbis virus replicons encoding Ag85A [26] or Ag85B [27] also showed some protective efficacy against *Mtb*, albeit when challenged at only 4 wk after parenteral vaccination, while mucosal immunogenicity of these replicons was not reported. T cell responses generated following parenteral administration of Vrep-Acr/Ag85B in the present study correlated with 4–5 fold reductions in bacterial loads in the lungs and spleens of mice following challenge with aerosolized *Mtb*, compared to those given control Vrep vaccine. Since challenge occurred at 10 wk post immunization, this represents durable, albeit relatively modest protection, that was not seen in mice vaccinated with conventional DNA vaccines encoding the same antigen fusion.

These findings, together with clear indications of their mucosal immunogenicity, suggest that Vrep are suitable for further testing, particularly as components of heterologous prime-boost vaccine strategies [59,60]. Parenteral immunization with Vrep encoding immunogenic *Mtb* antigens could be used to prime for potent immune responses, both in the spleen and in pulmonary tissues, that could be greatly enhanced by boosting with viral vectors, particularly via the respiratory tract. It is also likely that activation of innate and adaptive immune responses, including memory T cell responses at a local level, would best be achieved by priming directly via the respiratory route. We have begun studies to test the efficacy of nanoparticle-mediated pulmonary delivery of Vrep in animal models of TB infection, including direct comparisons of the Vrep-Acr/Ag85B fusion vaccine with Vrep expressing either of these antigens individually. Given the widespread use of the BCG vaccine, heterologous boosting of BCG-primed immunity will also likely be a key component of future TB vaccine strategies [60,61]. The lack of efficacy of a parenteral recombinant viral vaccine encoding Ag85A in BCG-immunized infants in a recent phase IIB trial has highlighted the need for greater understanding of the nature of protective immune responses against *Mtb* infection [62], including the role of local pulmonary immunity. A number of animal studies have demonstrated improved protection over BCG vaccine following mucosal rather than parenteral boosting [54,63,64], or when both BCG and a viral vector boost were given intranasally [65]. The mucosal immunogenicity of Vrep suggests that they may also be worthy of testing for their efficacy as boosters of BCG-primed pulmonary immunity.

Antigen-specific T cell responses to recall antigen are influenced by the maturation state of responding T cell subsets [66,67]. Varying the time interval between doses of vaccine, such as in prime–boost immunization, may help to determine whether the kinetics of T cell differentiation influence the immunogenicity and protective efficacy of immunization protocols. Thus, the interval between doses may be important for induction and possibly maturation of antigen-specific T cells, which could ultimately lead to improved vaccine efficacy. It was clear however, that extending the interval between Vrep immunizations from 3 to 6 wk in the present study enhanced neither the magnitude nor quality of T cell responses that were generated. Based on heterologous prime-boost studies [66,67], increasing the time interval even further than 6 wk could enhance vaccine immunogenicity, perhaps via optimizing stimulation of memory T cells generated following immune priming, although this was not the case in the present homologous vaccine delivery system. Given their immunogenicity however, it will be of interest to evaluate Vrep as priming vehicles in a variety of different prime-boost protocols, where the interval between Vrep priming and the administration of a heterologous booster vaccine may be an important determinant of efficacy.

The Acr/Ag85B fusion developed in these studies combines two immunogenic *Mtb* proteins in a single construct. Acr is widely regarded as a “latency” antigen and is certainly expressed at high levels during the non-replicating persistence phase of *Mtb* infection [39,68,69]. Recent work in this laboratory suggests that Acr, like Ag85B, is immunogenic soon after *Mtb* infection in the mouse model, and may therefore also represent a promising target for vaccination (Mehta and Ramsay, unpublished). Use of the Acr/Ag85B fusion may also hold promise as a component of a multi-stage gene-based vaccine, capable of eliciting immune responses against both acute and latent phases of TB infection. This concept was recently tested using a recombinant protein-based subunit fusion vaccine, comprising Ag85B and ESAT6 together with the TB latency antigen Rv2660c, that mediated significant protective efficacy against *Mtb* challenge in mice, when given either pre- or post-TB exposure [70].

In conclusion, our data suggest that Vrep encoding immunogenic *Mtb* proteins can induce long-lasting, antigen-specific T cell responses both in the spleen and in pulmonary tissues. These responses correlated with significant reduction of bacterial loads in these tissues following pulmonary challenge with *Mtb*. Studies are now underway in our laboratory to evaluate the mucosal and systemic immunogenicity and protective efficacy of Vrep, when incorporated as a priming vaccine in heterologous prime-boost immunization protocols, and also as a direct pulmonary immunogen.

## References

[1] Kaufmann SH. Future vaccination strategies against tuberculosis: thinking outside the box. Immunity 2010;33: 567–77. 10.1016/j.immuni.2010.09.015 21029966

[2] World Health Organization Global Tuberculosis Report 2014. WHO.

[3] McShane H. Vaccine strategies against tuberculosis. Swiss Med Wkly 2009;139: 156–60. doi: smw-12374 19152149 10.4414/smw.2009.12374

[4] Colditz GA, Brewer TF, Berkey CS, Wilson ME, Burdick E, Fineberg HV, et al Efficacy of BCG Vaccine in the Prevention of Tuberculosis: Meta-analysis of the Published Literature. JAMA 1994;271: 698–702. 8309034

[5] Kagina BMN, Abel B, Scriba TJ, Hughes EJ, Keyser A, Soares A, et al Specific T Cell Frequency and Cytokine Expression Profile Do Not Correlate with Protection against Tuberculosis after Bacillus Calmette-Guerin Vaccination of Newborns. Am J Respir Crit Care Med. 2010;182: 1073–79. 10.1164/rccm.201003-0334OC 20558627 PMC2970848

[6] Hesseling AC, Marais BJ, Gie RP, Schaaf HS, Fine PE, Godfrey-Faussett P. et al The risk of disseminated Bacille Calmette-Guerin (BCG) disease in HIV-infected children. Vaccine 2007; 25: 14–18. 16959383 10.1016/j.vaccine.2006.07.020

[7] Flynn JL, Goldstein MM, Triebold KJ, Koller B, Bloom BR. Major histocompatibility complex class I-restricted T cells are required for resistance to Mycobacterium tuberculosis infection. Proc Natl Acad Sci USA 1992;89: 12013–17. 1465432 10.1073/pnas.89.24.12013 PMC50688

[8] Cooper AM, Dalton DK, Stewart TA, Griffin JP, Russell DG, Orme IM, et al Disseminated tuberculosis in interferon gamma gene-disrupted mice. J Exp Med. 1993;178: 2243–47. 8245795 10.1084/jem.178.6.2243 PMC2191280

[9] Garcia I, Miyazaki Y, Marchal G, Lesslauer W, Vassalli P. High sensitivity of transgenic mice expressing soluble TNFR1 fusion protein to mycobacterial infections: Synergistic action of TNF and IFN-γ in the differentiation of protective granulomas. Eur J Immunol. 1997;27: 3182–90. 9464804 10.1002/eji.1830271215

[10] Scanga CA, Mohan VP, Yu K, Joseph H, Tanaka K, Chan J, et al Depletion of CD4(+) T cells causes reactivation of murine persistent tuberculosis despite continued expression of interferon gamma and nitric oxide synthase 2. J Exp Med. 2000;192: 347–58. 10934223 10.1084/jem.192.3.347 PMC2193220

[11] Britton WJ, Palendira U. Improving vaccines against tuberculosis. Immunol Cell Biol. 2003;81: 34–45. 12534944 10.1046/j.0818-9641.2002.01143.x

[12] Ulmer JB, Wahren B, Liu MA. Gene-based vaccines: recent technical and clinical advances. Trends Mol Med. 2006;12: 216–22. 16621717 10.1016/j.molmed.2006.03.007

[13] Dupuis M, Denis-Mize K, Woo C, Goldbeck C, Selby MJ, Chen M, et al Distribution of DNA vaccines determines their immunogenicity after intramuscular injection in mice. J Immunol. 2000;165: 2850–58. 10946318 10.4049/jimmunol.165.5.2850

[14] Leitner WW, Restifo NP. DNA vaccines and apoptosis: to kill or not to kill? J Clin Invest. 2003;112: 22–24. 12840054 10.1172/JCI19069 PMC162296

[15] Powell K. DNA vaccines-back in the saddle again? Nat Biotechnol. 2004;22: 799–801. 15229530 10.1038/nbt0704-799 PMC7097118

[16] Kalams SA, Parker S, Jin X, Elizaga M, Metch B, Wang M, et al Safety and immunogenicity of an HIV-1 gag DNA vaccine with or without IL-12 and/or IL-15 plasmid cytokine adjuvant in healthy, HIV-1 uninfected adults. PLoS One 2012;7: e29231 10.1371/journal.pone.0029231 22242162 PMC3252307

[17] Giri M, Ugen KE, Weiner DB. DNA vaccines against human immunodeficiency virus type 1 in the past decade. Clin Microbiol Rev. 2004;17: 370–89. 15084506 10.1128/CMR.17.2.370-389.2004 PMC387404

[18] Gilbert SC, Moorthy VS, Andrews L, Pathan AA, McConkey SJ, Vuola JM, et al Synergistic DNA-MVA prime-boost vaccination regimes for malaria and tuberculosis. Vaccine 24: 2006; 4554–61. 16150517 10.1016/j.vaccine.2005.08.048

[19] Wang S, Kennedy JS, West K, Montefiori DC, Coley S, Lawrence J, et al Cross-subtype antibody and cellular immune responses induced by a polyvalent DNA prime-protein boost HIV-1 vaccine in healthy human volunteers. Vaccine 2008;26: 3947–57. 18724414 10.1016/j.vaccine.2007.12.060 PMC3743087

[20] Li Z, Zhang H, Fan X, Zhang Y, Huang J, Liu Q, et al DNA electroporation prime and protein boost strategy enhances humoral immunity of tuberculosis DNA vaccines in mice and non-human primates. Vaccine 2006;24: 4565–68. 16154246 10.1016/j.vaccine.2005.08.021

[21] Rosati M, Valentin A, Jalah R, Patel V, von Gegerfelt A, Bergamaschi C, et al Increased immune responses in rhesus macaques by DNA vaccination combined with electroporation. Vaccine 2008;26: 5223–29. 10.1016/j.vaccine.2008.03.090 18468743 PMC7263013

[22] Young SL, Slobbe LJ, Peacey M, Gilbert SC, Buddle BM, de Lisle GW, et al Immunogenicity and protective efficacy of mycobacterial DNA vaccines incorporating plasmid-encoded cytokines against Mycobacterium bovis. Immunol Cell Biol. 2010;88: 651–57. 10.1038/icb.2010.25 20231853

[23] Lu S, Wang S, Grimes-Serrano JM. Current progress of DNA vaccine studies in humans. Expert Rev Vaccines 2008;7: 175–91. 10.1586/14760584.7.2.175 18324888

[24] Ljungberg K, Whitmore AC, Fluet ME, Moran TP, Shabman RS, Collier ML, et al Increased Immunogenicity of a DNA-Launched Venezuelan Equine Encephalitis Virus-Based Replicon DNA Vaccine. J Virol. 2007; 81: 13412–23. 17913817 10.1128/JVI.01799-07 PMC2168848

[25] Berglund P, Smerdou C, Fleeton MN, Tubulekas I, Liljestrom P. Enhancing immune responses using suicidal DNA vaccines. Nat Biotechnol. 1998;16: 562–65. 9624688 10.1038/nbt0698-562

[26] Kirman JR, Turon T, Su H, Li A, Kraus C, Polo JM, et al Enhanced immunogenicity to Mycobacterium tuberculosis by vaccination with an alphavirus plasmid replicon expressing antigen 85A. Infect Immun. 2003;71: 575–79. 12496215 10.1128/IAI.71.1.575-579.2003 PMC143413

[27] Derrick SC, Yang AL, Morris SL. Vaccination with a Sindbis virus-based DNA vaccine expressing antigen 85B induces protective immunity against Mycobacterium tuberculosis. Infect Immun. 2005;73: 7727–35. 16239577 10.1128/IAI.73.11.7727-7735.2005 PMC1273836

[28] Atkins GJ, Fleeton MN, Sheahan BJ. Therapeutic and prophylactic applications of alphavirus vectors. Expert Rev Mol Med. 2007;10: e33. 10.1017/S1462399408000859 19000329

[29] Knudsen ML, Mbewe-Mvula A, Rosario M, Johansson DX, Kakoulidou M, Bridgeman A, et al Superior induction of T cell responses to conserved HIV-1 regions by electroporated alphavirus replicon DNA compared to that with conventional plasmid DNA vaccine. J Virol. 2012;86: 4082–90. 10.1128/JVI.06535-11 22318135 PMC3318663

[30] Thompson JM, Whitmore AC, Konopka JL, Collier ML, Richmond EM, Davis NL, et al Mucosal and systemic adjuvant activity of alphavirus replicon particles. Proc Natl Acad Sci USA 2006;103: 3722–27. 16505353 10.1073/pnas.0600287103 PMC1383499

[31] Thompson JM, Whitmore AC, Staats HF, Johnston RE. Alphavirus replicon particles acting as adjuvants promote CD8+ T cell responses to co-delivered antigen. Vaccine 2008;26: 4267–75. 10.1016/j.vaccine.2008.05.046 18582997 PMC3608392

[32] Gardner JP, Frolov I, Perri S, Ji Y, MacKichan ML, Chen M, et al Infection of human dendritic cells by a sindbis virus replicon vector is determined by a single amino acid substitution in the E2 glycoprotein. J Virol. 2000;74: 11849–57. 11090185 10.1128/jvi.74.24.11849-11857.2000 PMC112468

[33] MacDonald GH, Johnston RE. Role of dendritic cell targeting in Venezuelan equine encephalitis virus pathogenesis. J Virol. 2000;74: 914–22. 10623754 10.1128/jvi.74.2.914-922.2000 PMC111612

[34] Barry G, Breakwell L, Fragkoudis R, Attarzadeh-Yazdi G, Rodriguez-Andres J, Fazakerley JK, et al PKR acts early in infection to suppress Semliki Forest virus production and strongly enhances the type I interferon response. J Gen Virol. 2009;90: 1382–91. 10.1099/vir.0.007336-0 19264662 PMC2885058

[35] Schulz O, Diebold SS, Chen M, Naslund TI, Nolte MA, Alexopoulou L, et al Toll-like receptor 3 promotes cross-priming to virus-infected cells. Nature 2005;433: 887–92. 15711573 10.1038/nature03326

[36] Barry G, Fragkoudis R, Ferguson MC, Lulla A, Merits A, Kohl A, et al Semliki forest virus-induced endoplasmic reticulum stress accelerates apoptotic death of mammalian cells. J Virol. 2010;84: 7369–77. 10.1128/JVI.02310-09 20427528 PMC2898233

[37] Li ML, Stollar V. Alphaviruses and apoptosis. Int Rev Immunol. 2004;23: 7–24. 14690853 10.1080/08830180490265529

[38] Belisle JT, Vissa VD, Sievert T, Takayama K, Brennan PJ, Besra GS, et al Role of the major antigen of Mycobacterium tuberculosis in cell wall biogenesis. Science 1997;276: 1420–22. 9162010 10.1126/science.276.5317.1420

[39] Wayne LG, Sohaskey CD. Nonreplicating persistence of Mycobacterium Tuberculosis. Ann Rev Microbiol. 2001;55: 139–63. 11544352 10.1146/annurev.micro.55.1.139

[40] Purkayastha A, McCue LA, McDonough KA. Identification of a Mycobacterium tuberculosis Putative Classical Nitroreductase Gene Whose Expression Is Coregulated with That of the acr Gene within Macrophages, in Standing versus Shaking Cultures, and under Low Oxygen Conditions. Infect Immun. 2002;70: 1518–29. 11854240 10.1128/IAI.70.3.1518-1529.2002 PMC127740

[41] Auten MW, Huang W, Dai G, Ramsay AJ. CD40 ligand enhances immunogenicity of vector-based vaccines in immunocompetent and CD4+ T cell deficient individuals. Vaccine 2012;30: 2768–77. 10.1016/j.vaccine.2012.02.020 22349523 PMC3313012

[42] Pushko P, Parker M, Ludwig GV, Davis NL, Johnston RE, Smith JF, et al Replicon-helper systems from attenuated Venezuelan equine encephalitis virus: expression of heterologous genes in vitro and immunization against heterologous pathogens in vivo. Virology 1997;239: 389–401. 9434729 10.1006/viro.1997.8878

[43] Balasuriya UB, Heidner HW, Hedges JF, Williams JC, Davis NL, Johnston RE, et al Expression of the two major envelope proteins of equine arteritis virus as a heterodimer is necessary for induction of neutralizing antibodies in mice immunized with recombinant Venezuelan equine encephalitis virus replicon particles. J Virol, 2000;74: 10623–30. 11044106 10.1128/jvi.74.22.10623-10630.2000 PMC110936

[44] Yin J, Gardner CL, Burke CW, Ryman KD, Klimstra WB. Similarities and differences in antagonism of neuron alpha/beta interferon responses by Venezuelan equine encephalitis and Sindbis alphaviruses. J Virol. 2009;83: 10036–47. 10.1128/JVI.01209-09 19641001 PMC2748036

[45] Zhang Y, Burke CW, Ryman KD, Klimstra WB. Identification and characterization of interferon-induced proteins that inhibit alphavirus replication. J Virol. 2007;81: 11246–55. 17686841 10.1128/JVI.01282-07 PMC2045553

[46] Vordermeier HM, Harris DP, Lathigra R, Roman E, Moreno C, Ivanyi J. Recognition of peptide epitopes of the 16,000 MW antigen of Mycobacterium tuberculosis by murine T cells. Immunology 1993;80: 6–12. 7503946 PMC1422108

[47] Roupie V, Romano M, Zhang L, Korf H, Lin MY, Franken KL, et al Immunogenicity of eight dormancy regulon-encoded proteins of Mycobacterium tuberculosis in DNA-vaccinated and tuberculosis-infected mice. Infect Immun. 2007;75: 941–49. 17145953 10.1128/IAI.01137-06 PMC1828490

[48] D'Souza S, Rosseels V, Romano M, Tanghe A, Denis O, Jurion F, et al Mapping of murine Th1 helper T-Cell epitopes of mycolyl transferases Ag85A, Ag85B, and Ag85C from Mycobacterium tuberculosis. Infect Immun. 2003;71: 483–93. 12496199 10.1128/IAI.71.1.483-493.2003 PMC143283

[49] Radosevic K, Wieland CW, Rodriguez A, Weverling GJ, Mintardjo R, Gillissen G, et al Protective immune responses to a recombinant adenovirus type 35 tuberculosis vaccine in two mouse strains: CD4 and CD8 T-cell epitope mapping and role of gamma interferon. Infect Immun. 2007;75: 4105–15. 17526747 10.1128/IAI.00004-07 PMC1951991

[50] Belyakov IM, Ahlers JD. What role does the route of immunization play in the generation of protective immunity against mucosal pathogens? J Immunol. 2009;183: 6883–92. 10.4049/jimmunol.0901466 19923474

[51] Haan L, Verweij WR, Holtrop M, Brands R, van Scharrenburg GJ, Palache AM, et al Nasal or intramuscular immunization of mice with influenza subunit antigen and the B subunit of Escherichia coli heat-labile toxin induces IgA- or IgG-mediated protective mucosal immunity. Vaccine 2001;19: 2898–907. 11282201 10.1016/s0264-410x(00)00556-9

[52] Darrah PA, Patel DT, De Luca PM, Lindsay RWB, Davey DF, Flynn BJ, et al Multifunctional TH1 cells define a correlate of vaccine-mediated protection against Leishmania major. Nat Med. 2007;13: 843–50. 17558415 10.1038/nm1592

[53] Genesca M, Rourke T, Li J, Bost K, Chohan B, McChesney MB, et al Live Attenuated Lentivirus Infection Elicits Polyfunctional Simian Immunodeficiency Virus Gag-Specific CD8+ T Cells with Reduced Apoptotic Susceptibility in Rhesus Macaques that Control Virus Replication after Challenge with Pathogenic SIVmac239. J Immunol. 2007;179: 4732–40. 17878372 10.4049/jimmunol.179.7.4732 PMC3401023

[54] Forbes EK, Sander C, Ronan EO, McShane H, Hill AVS, Beverley PC, et al Multifunctional, High-Level Cytokine-Producing Th1 Cells in the Lung, but Not Spleen, Correlate with Protection against Mycobacterium tuberculosis Aerosol Challenge in Mice. J Immunol. 2008;181: 4955–64. 18802099 10.4049/jimmunol.181.7.4955 PMC2867031

[55] Kalsdorf B, Scriba TJ, Wood K, Day CL, Dheda K, Dawson R, et al HIV-1 infection impairs the bronchoalveolar T-cell response to mycobacteria. Am J Respir Crit Care Med. 2009;180: 1262–70. 10.1164/rccm.200907-1011OC 19797156 PMC2796736

[56] Sutherland JS, Adetifa IM, Hill PC, Adegbola RA, Ota MO. Pattern and diversity of cytokine production differentiates between Mycobacterium tuberculosis infection and disease. Eur J Immunol. 2009;39: 723–29. 10.1002/eji.200838693 19224636

[57] Wilkinson KA, Wilkinson RJ. Polyfunctional T cells in human tuberculosis. Eur J Immunol. 2010;40: 2139–42. 10.1002/eji.201040731 20853500

[58] Beverley PC, Sridhar S, Lalvani A, Tchilian EZ. Harnessing local and systemic immunity for vaccines against tuberculosis. Mucosal Immunol. 2014;7: 20–26. 10.1038/mi.2013.99 24253104

[59] Ramshaw IA, Ramsay AJ. The prime-boost strategy: exciting prospects for improved vaccination. Immunol Today 2000;21: 163–65. 10740236 10.1016/s0167-5699(00)01612-1

[60] Dalmia N, Ramsay AJ. Prime-boost approaches to tuberculosis vaccine development. Expert Rev Vaccines 2012;11: 1221–33. 10.1586/erv.12.94 23176655 PMC3572762

[61] McShane H. Tuberculosis vaccines: beyond bacille Calmette-Guerin. Philos Trans R Soc Lond B Biol Sci. 2011;366: 2782–89. 10.1098/rstb.2011.0097 21893541 PMC3146779

[62] Tameris MD, Hatherill M, Landry BS, Scriba TJ, Snowden MA, Lockhart S, et al Safety and efficacy of MVA85A, a new tuberculosis vaccine, in infants previously vaccinated with BCG: a randomised, placebo-controlled phase 2b trial. Lancet 2013;381: 1021–28. 23391465 10.1016/S0140-6736(13)60177-4 PMC5424647

[63] Jeyanathan M, Damjanovic D, Shaler CR, Lai R, Wortzman M, Yin C, et al Differentially imprinted innate immunity by mucosal boost vaccination determines antituberculosis immune protective outcomes, independent of T-cell immunity. Mucosal Immunol. 2013;6: 612–25. 10.1038/mi.2012.103 23131783

[64] Xing Z, McFarland CT, Sallenave JM, Izzo A, Wang J, McMurray DN. Intranasal Mucosal Boosting with an Adenovirus-Vectored Vaccine Markedly Enhances the Protection of BCG-Primed Guinea Pigs against Pulmonary Tuberculosis. PLoS One 2009;4: e5856 10.1371/journal.pone.0005856 19516906 PMC2689939

[65] Goonetilleke NP, McShane H, Hannan CM, Anderson RJ, Brookes RH, Hill AV. Enhanced Immunogenicity and Protective Efficacy Against Mycobacterium tuberculosis of Bacille Calmette-Guerin Vaccine Using Mucosal Administration and Boosting with a Recombinant Modified Vaccinia Virus Ankara. J Immunol. 2003;171: 1602–9. 12874255 10.4049/jimmunol.171.3.1602

[66] Brice GT, Dobano C, Sedegah M, Stefaniak M, Graber NL, Campo JJ, et al Extended immunization intervals enhance the immunogenicity and protective efficacy of plasmid DNA vaccines. Microbes Infect. 2007;9: 1439–46. 17913540 10.1016/j.micinf.2007.07.009

[67] Weiss WR, Kumar A, Jiang G, Williams J, Bostick A, Conteh S, et al Protection of rhesus monkeys by a DNA prime/poxvirus boost malaria vaccine depends on optimal DNA priming and inclusion of blood stage antigens. PLoS One 2007;2: e1063 17957247 10.1371/journal.pone.0001063 PMC2031826

[68] Yuan Y, Crane DD, Barry CE 3rd. Stationary phase-associated protein expression in Mycobacterium tuberculosis: function of the mycobacterial alpha-crystallin homolog. J Bacteriol. 1996;178: 4484–92. 8755875 10.1128/jb.178.15.4484-4492.1996 PMC178214

[69] Shi L, Jung YJ, Tyagi S, Gennaro ML, North RJ. Expression of Th1-mediated immunity in mouse lungs induces a Mycobacterium tuberculosis transcription pattern characteristic of nonreplicating persistence. Proc Natl Acad Sci USA 2003;100: 241–46. 12506197 10.1073/pnas.0136863100 PMC140939

[70] Aagaard C, Hoang T, Dietrich J, Cardona PJ, Izzo A, Dolganov G, et al A multistage tuberculosis vaccine that confers efficient protection before and after exposure. Nat Med. 2011;17: 189–94. 10.1038/nm.2285 21258338

